# Supplementary material for: Experimental and computational methods for allelic imbalance analysis from single-nucleus RNA-seq data
Source: Genome Biol. 2026 Apr 11;27:167. doi: 10.1186/s13059-026-04062-6 (PMC13185290; doi:10.1186/s13059-026-04062-6)
Supplement: Supplementary file 2 — Additional file 2: Supplementary Figures. Figure S1. ASE with or without intron-aligned UMIs. Figures S2. Read length effect on power in each cell type. Figure S3. Isoform-level analysis. Figure S4. Long-read error rates. Figure S5. Hybrid selection metrics. Figure S6. Additional PD sample analysis. Figure S7. Sierra peak analysis. Figure S8. Allelic imbalance vs. eQTL analysis for different statistics. Figure S9. Differential allelic imbalance. Figure S10. Experimental design. Figure S11. Detailed computational pipeline. [file 13059_2026_4062_MOESM2_ESM.pdf]

Additional file 2:

Supplementary Information for Simmons et al. "Experimental and computational methods for allelic imbalance analysis from single-nucleus RNA-seq data" Genome Biology 2025

Supplementary Figures 1 - 11

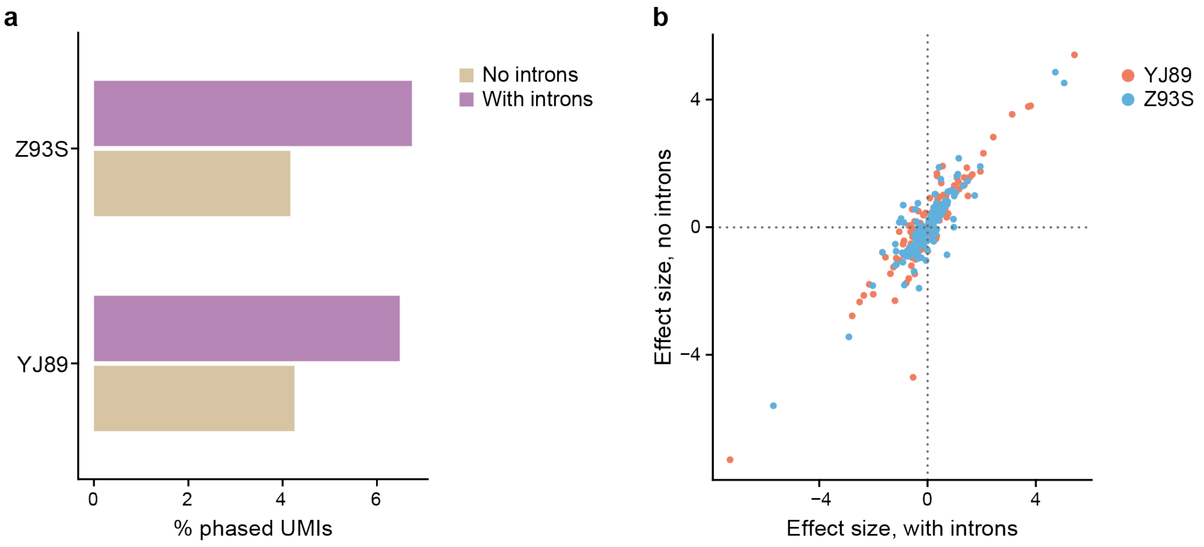

Fig. S1 ASE with or without intron-aligned UMIs

a Comparison of the percentage of UMIs that are phased in each sample either including or excluding introns. **b** Comparison of the estimated allelic imbalance effect size for genes that are significant in either analysis with or without introns for each sample.

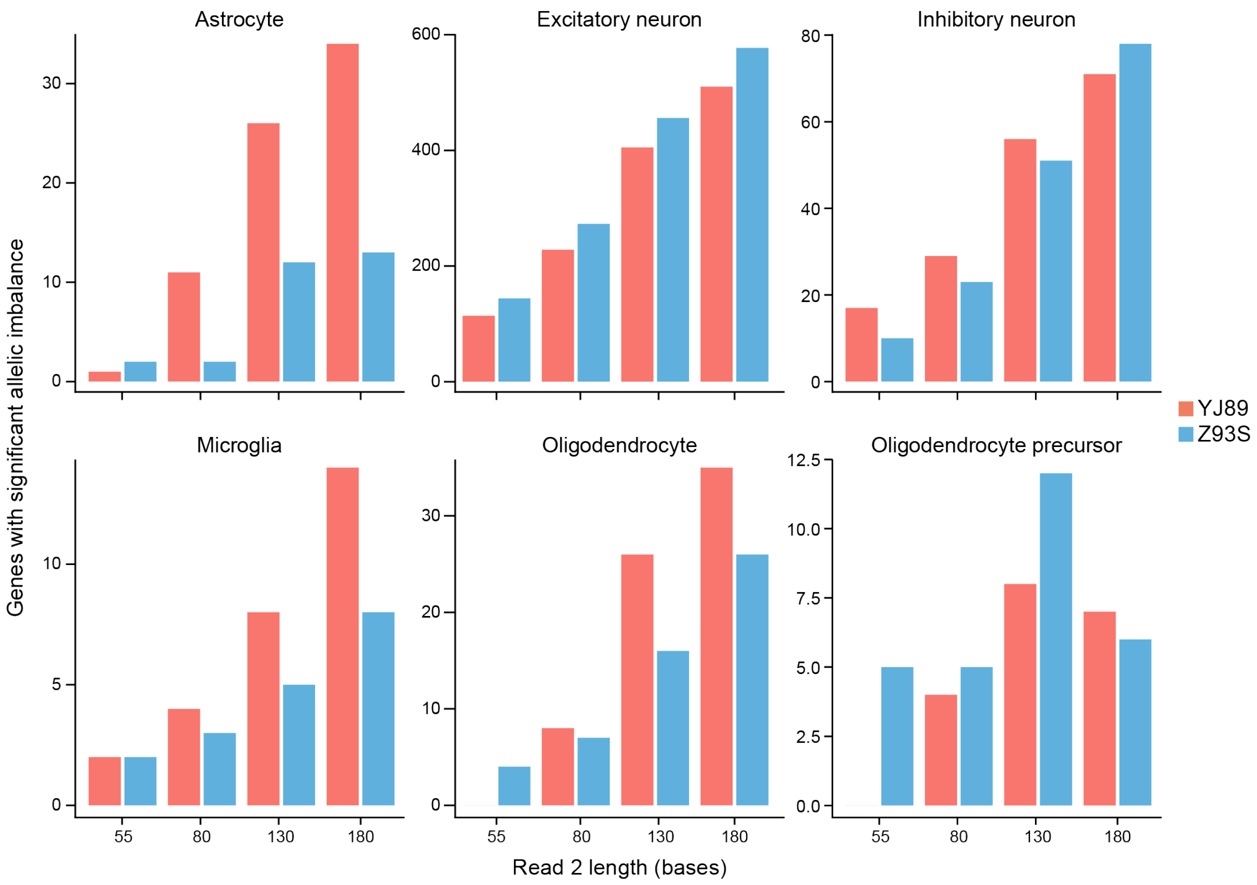

Fig. S2 Read length effect on power in each cell type.

Shown for each cell type is the number of genes with significant allelic imbalance for each sample at different read lengths.

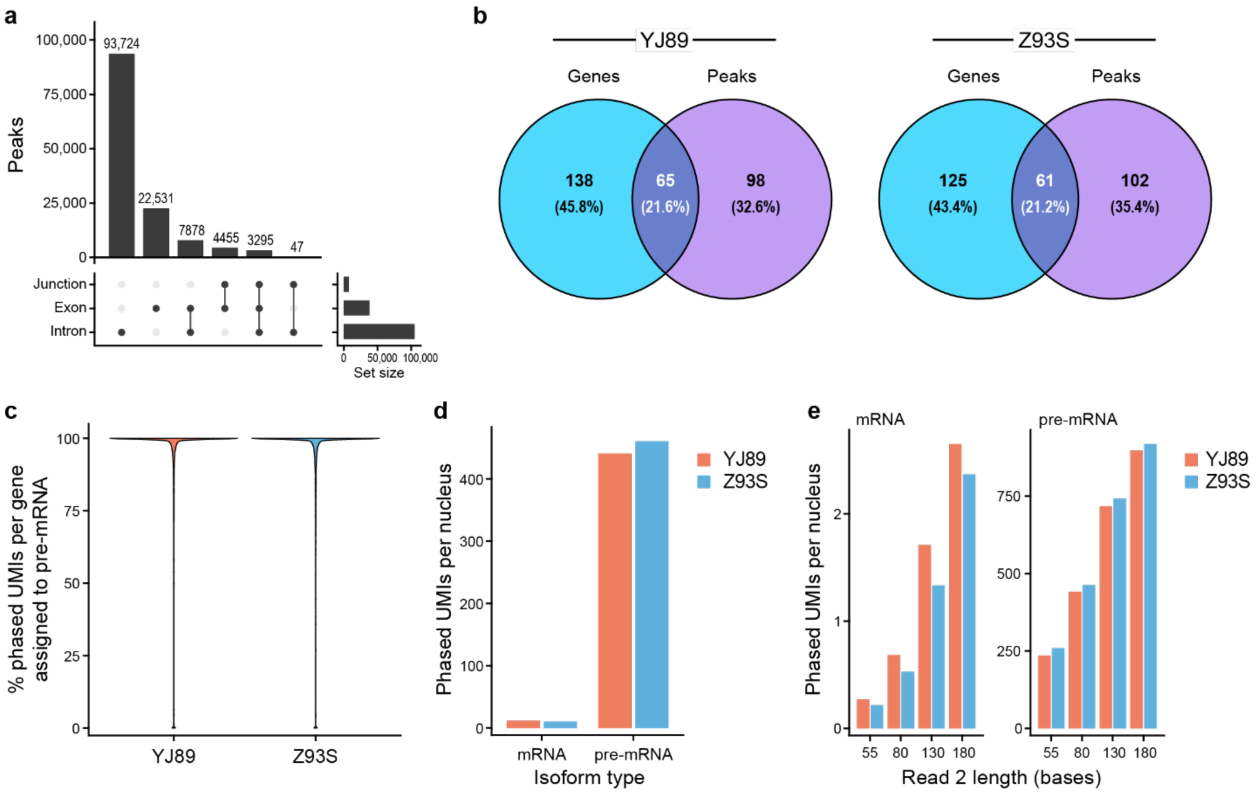

Fig. S3 Isoform-level analysis.

a UpSet plot of the number of peaks assigned to a genomic region according to Sierra's peak annotation. Exon includes 5' UTR and 3' UTR peaks. **b** Venn diagram for each sample showing overlap between genes with significant allelic imbalance and genes with at least one Sierra peak with significant allelic imbalance. **c** Violin plot showing the percentage of phased UMIs coming from the unspliced pre-mRNA transcript for each gene with at least 10 phased UMIs in the isoform level analysis for each sample. **d** Phased UMIs per cell recovered with isoform-level analysis of the MAS-Seq data for both spliced and unspliced reads. **e** Phased UMIs per cell recovered with isoform-level analysis of the short-read data for spliced and unspliced reads with varying lengths of read 2.

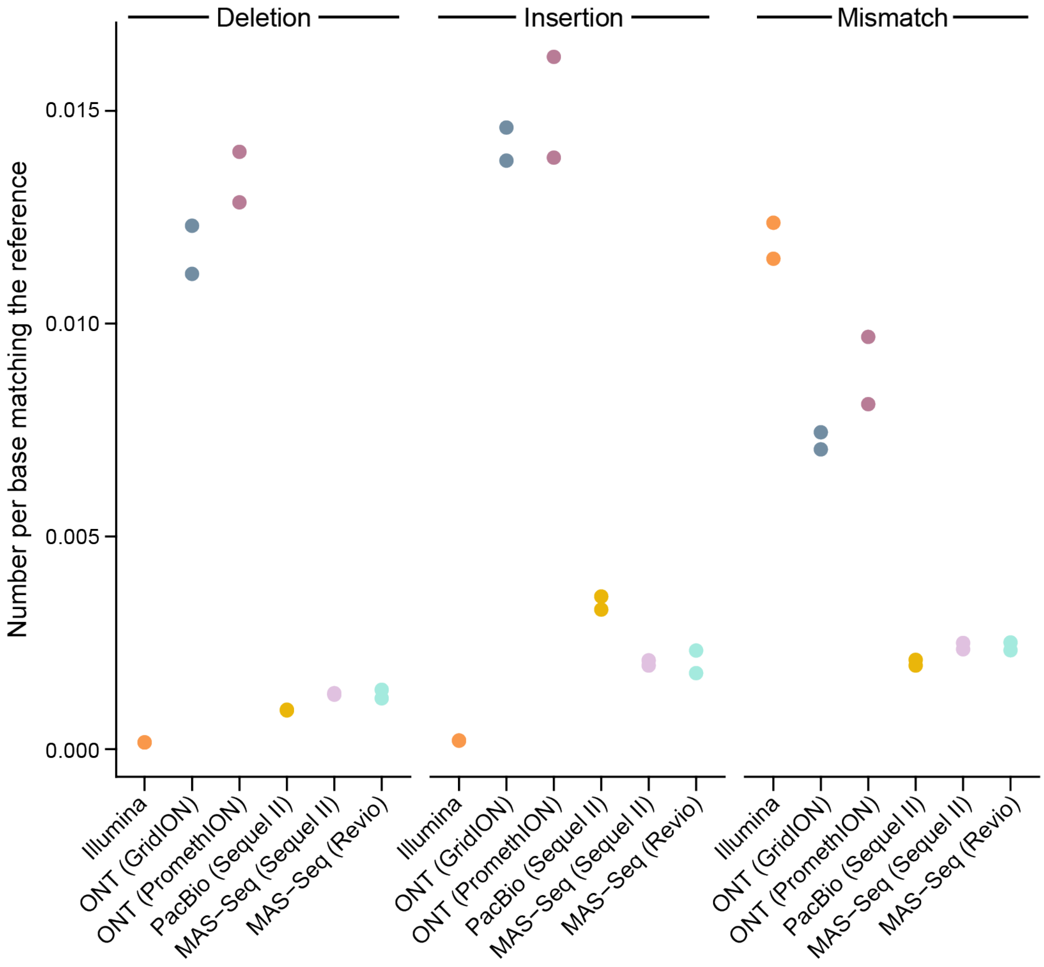

Fig. S4. Long-read error rates

For each sequencing technology, shown is the frequency a base disagreed with the reference genome, with either an insertion, deletion, or mismatch. We counted matches (positions were the read matches the reference genome), mismatches (positions where the read maps to the genome but the nucleotide does not match the reference), insertions (locations with an insertion in the read relative to the reference genome), and deletions (locations with a deletion in the read relative to the reference genome, excluding splicing events). We then calculated the number of insertions, deletions, and mismatches divided by the number of matches and plotted the results.

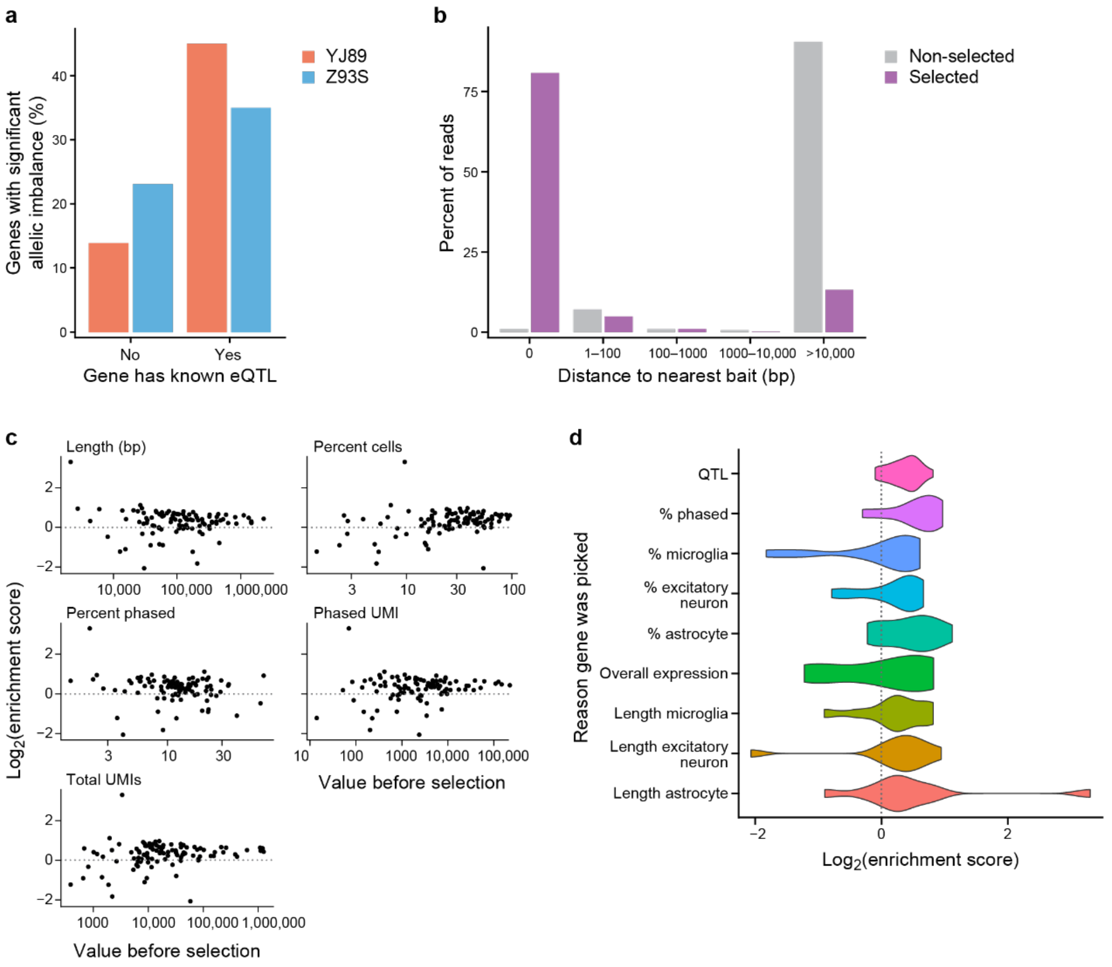

Fig. S5 Hybrid selection metrics.

a Percentage of targeted genes with significant allelic imbalance shown for genes with or without a known eQTL. **b** Distance of uniquely mapped reads from hybrid selection baits with and without selection**. c** Comparison of the $\log_2$ of the enrichment score (phased UMIs after selection / phased UMIs before selection) for each selected gene to different metrics for each gene without selection. Total UMI: total UMIs assigned to that gene, Phased UMI: total phased UMIs assigned to that gene, Length: length of the gene in bp, Percentage Phased: percentage of Phased UMI divided by Total UMI, and Percentage Cells: percentage of cells expressing that gene. **d** Violin plots of the enrichment score for genes chosen by each criterion (see “Methods”).

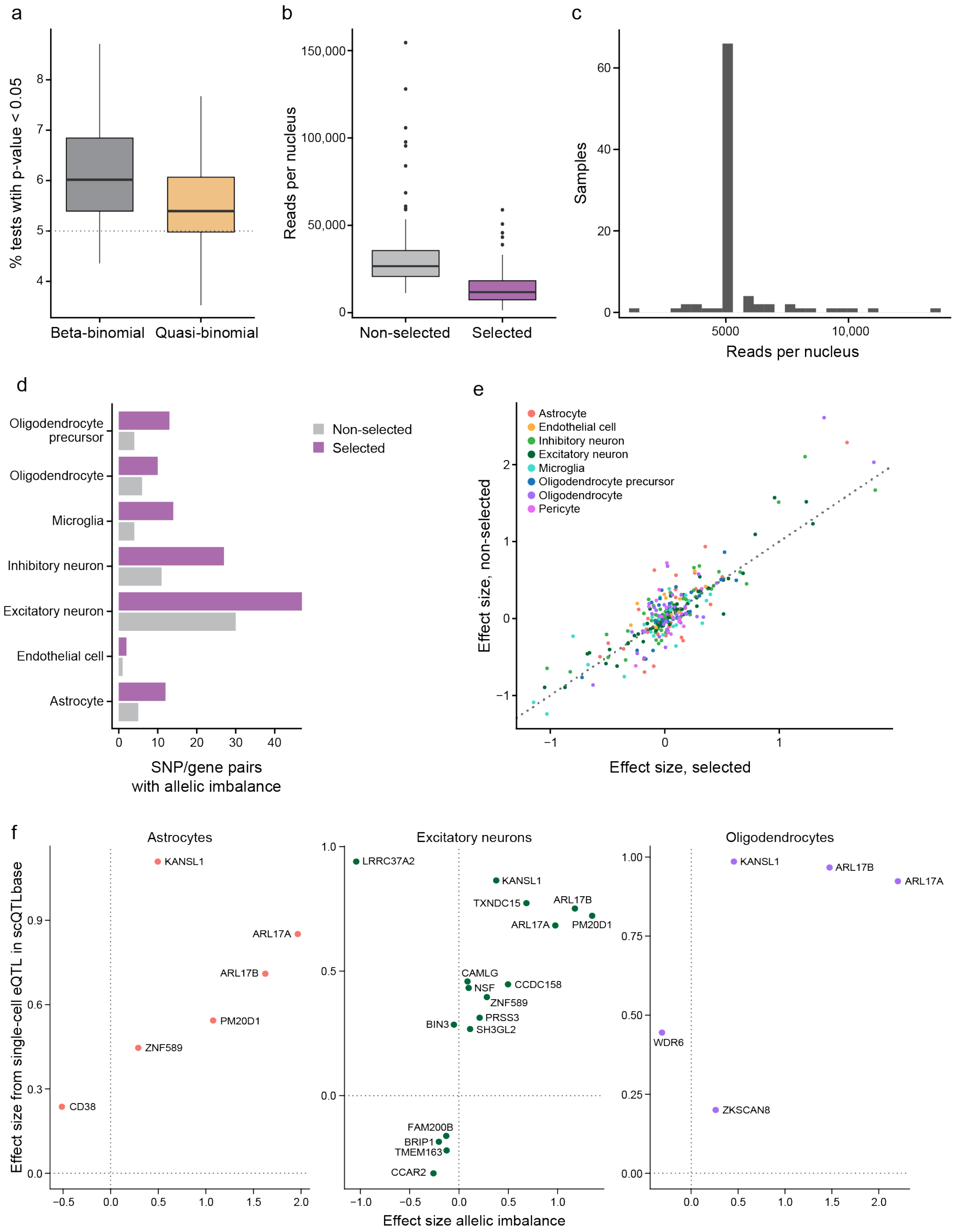

Fig. S6 Additional PD sample analysis.

a Box plots of false positive rate (FPR) analysis for beta-binomial *vs.* quasi-binomial pseudobulk methods (x-axis). Results of random permutation (100 times, glutamatergic neurons data) of the alternate *vs.* reference allele for each individual to generate a dataset with no true allelic imbalance. Shown for each permutation is the percentage of comparisons with p-value < 0.05, which should be around 5% if the method controls the FPR. Boxplots denote the medians and the interquartile ranges (IQRs). The whiskers of each boxplot are the lowest datum still within 1.5 IQR of the lower quartile and the highest datum still within 1.5 IQR of the upper quartile. **b** Boxplots of sequence coverage without selection and with selection. **c** Histogram of sequence coverage with downsampling. Overall, there is an average of 5,365 reads per cell in this dataset. Because the downsampling was based on the number of nuclei reported by Cell Ranger and these results were based on the number of nuclei in the final Seurat object (a smaller number of nuclei), some samples had more than 5,000 reads per nucleus. Those with fewer than 5,000 reads per nucleus were not downsampled. **d** Comparison of the number of SNP/gene pairs with significant allelic imbalance in each cell type for downsampled selected data and non-downsampled non-selected data. **e** Comparison of effect size estimates for each targeted gene and each cell type in the data with and without selection. **f** Comparison of allelic imbalance results (x-axis) to single-cell eQTL results (y-axis) from the scQTLbase database for astrocytes, excitatory neurons, and oligodendrocytes.

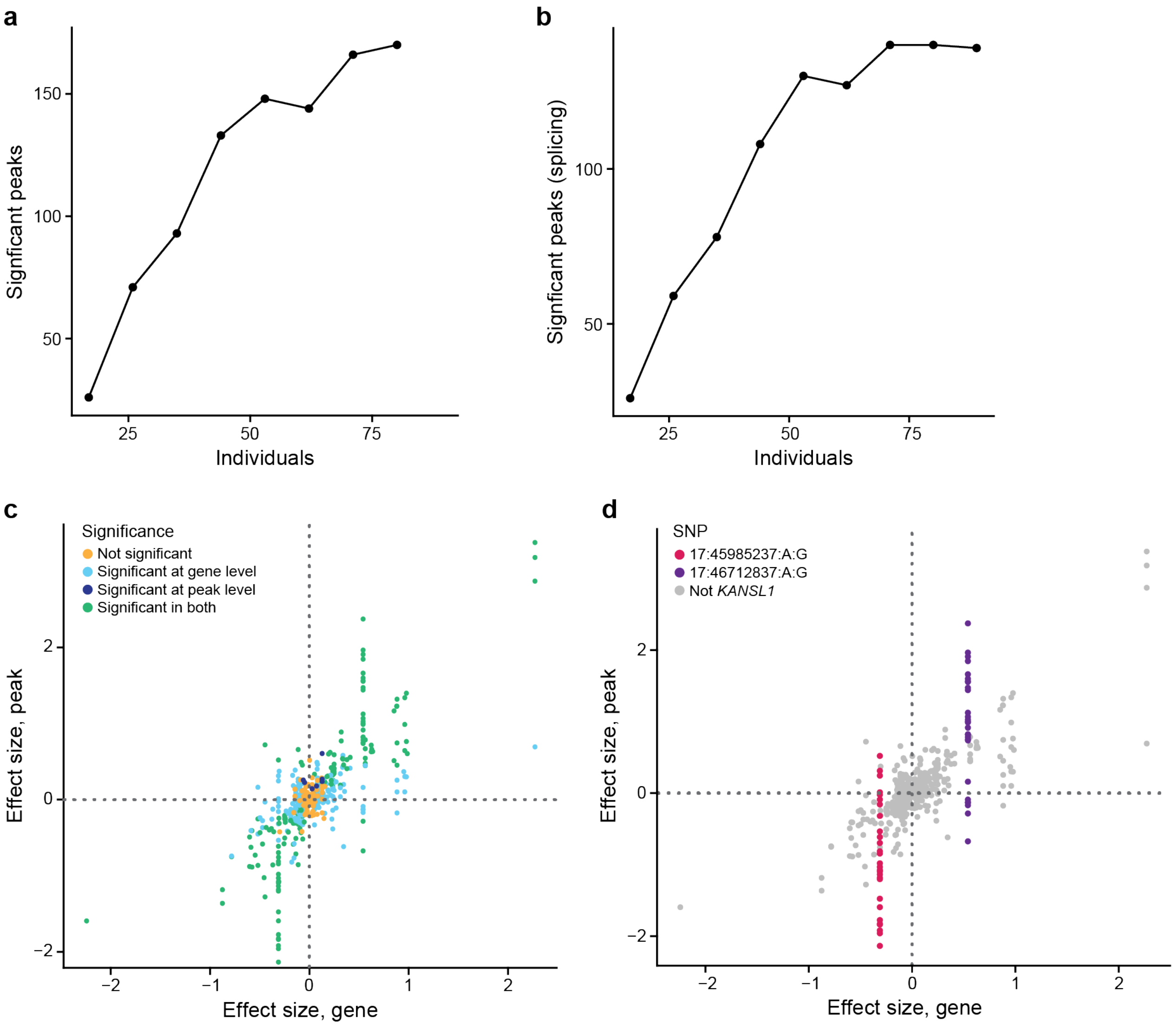

Fig. S7 Sierra peak analysis

Effects of downsampling the number of individuals on Sierra peaks with significant allelic imbalance (**a**) and Sierra peaks with significant allelic imbalance relative to the other peaks in the same genes (**b**). **c** Estimated allelic imbalance for each peak *vs.* estimated allelic imbalance for the associated gene. **d** As in **c**, highlighting *KANSL1* peaks.

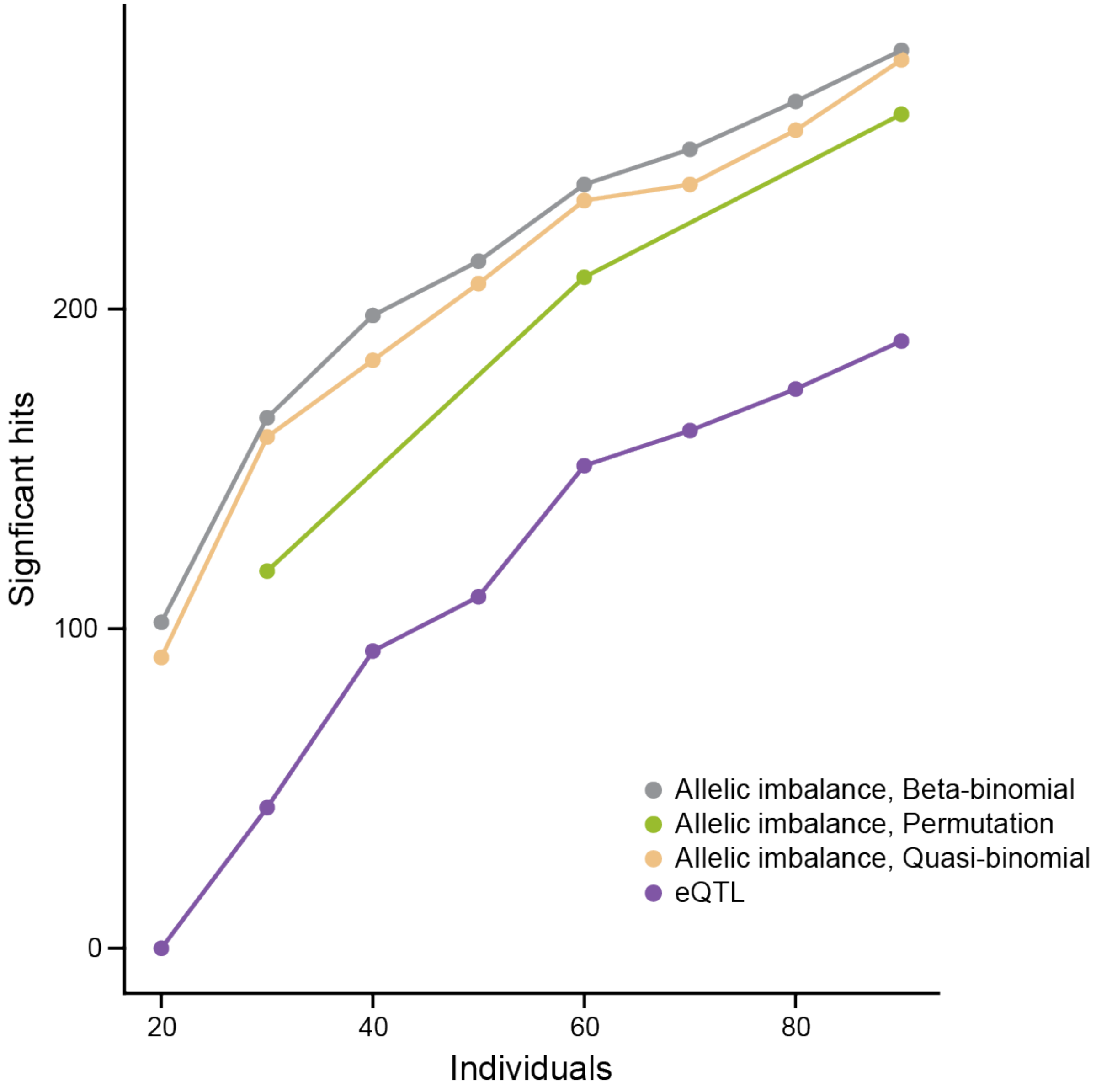

Fig. S8 Allelic imbalance *vs.* eQTL analysis for different statistics

Comparison of significant SNP/gene pairs (y-axis) detected by eQTL or allelic imbalance analysis with different number of individuals sampled (x-axis) and with different methods for allelic imbalance. Significant hits had a Benjamini–Hochberg-corrected p-value < 0.05. Detection of more SNP/gene pairs indicates greater power.

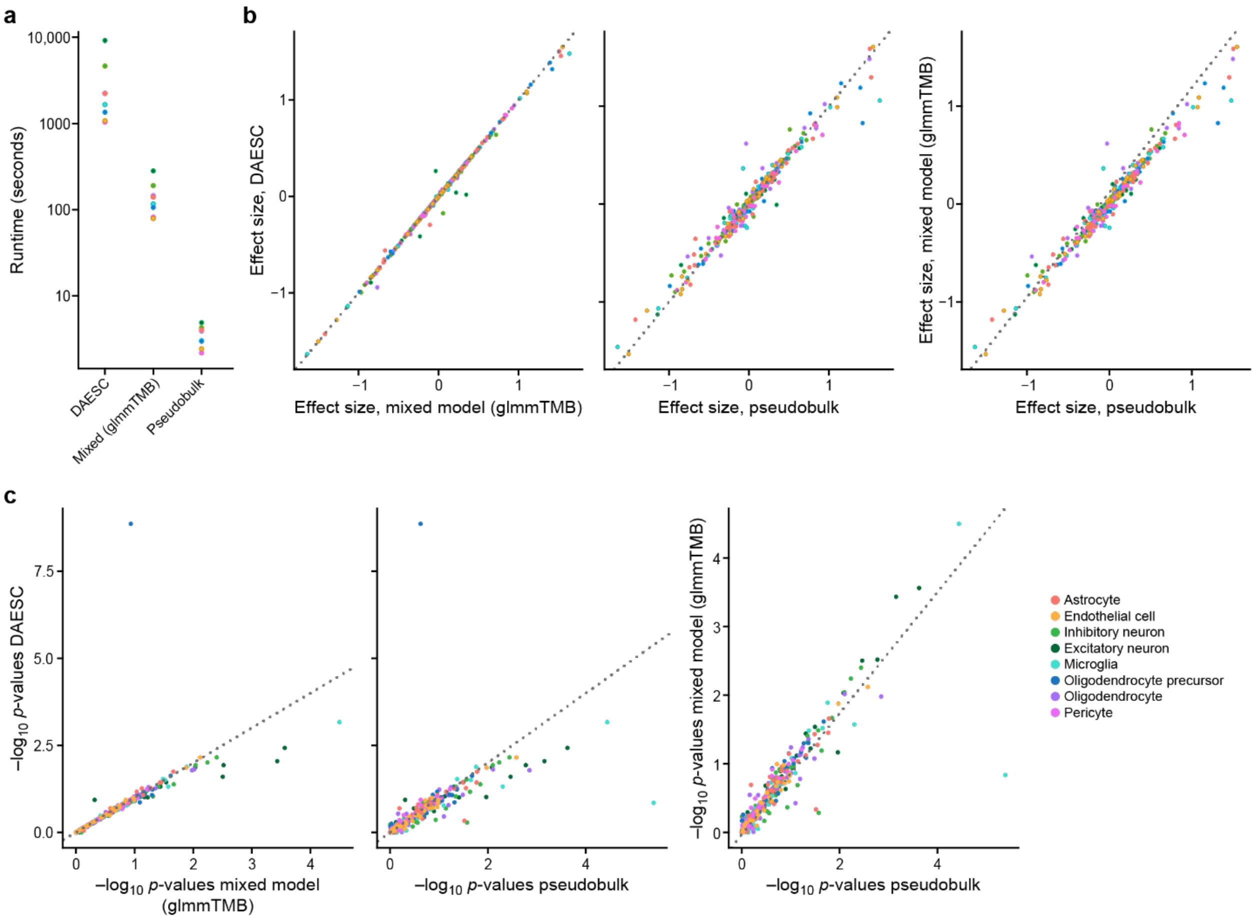

Fig. S9 Differential allelic imbalance.

a Runtime of each method for each cell type. Scatter plots comparing the estimated effect size (**b**) and the negative $\log_{10}$ p-values (**c**) from differential allelic imbalance analysis (PD *vs.* healthy controls) for each method in each cell type. All analyses used the PD data with selection and no downsampling.

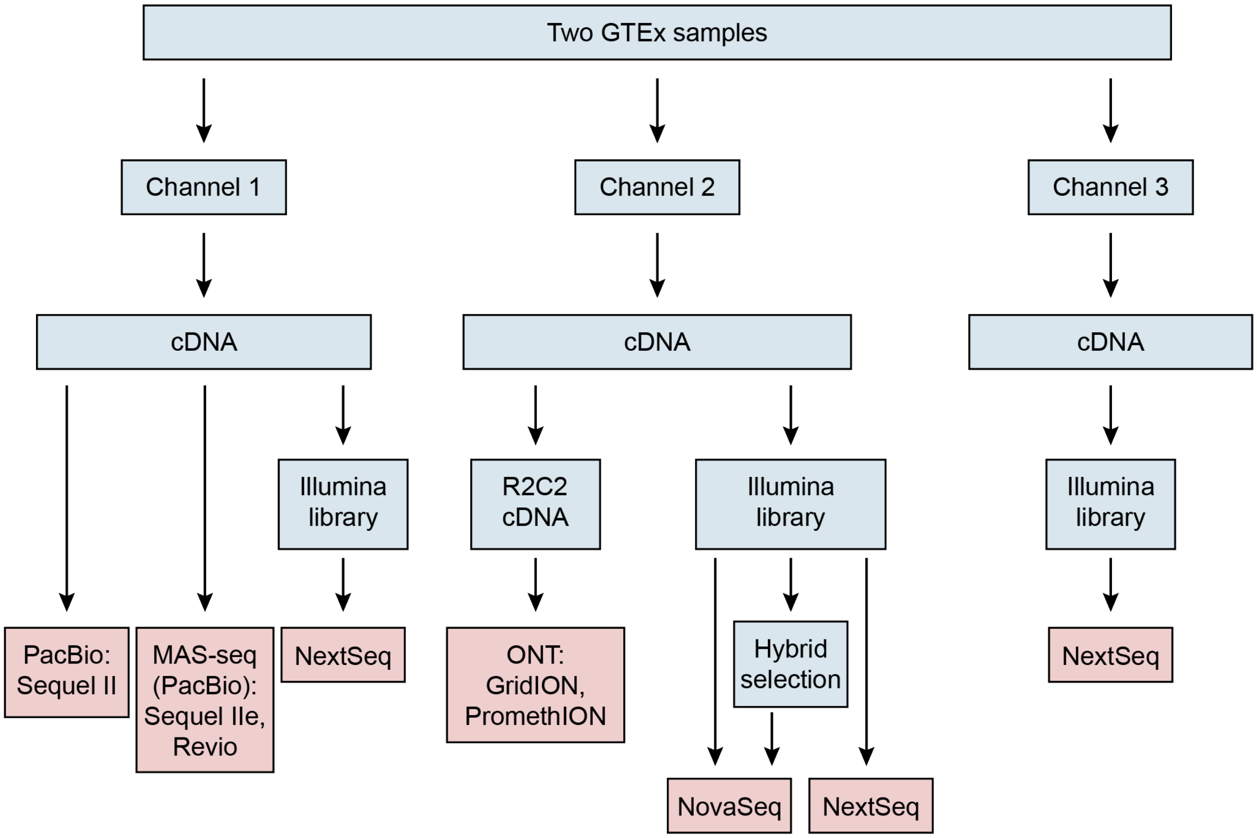

Fig. S10 Experimental design.

Schematic showing processing of each library with the GTEx samples.

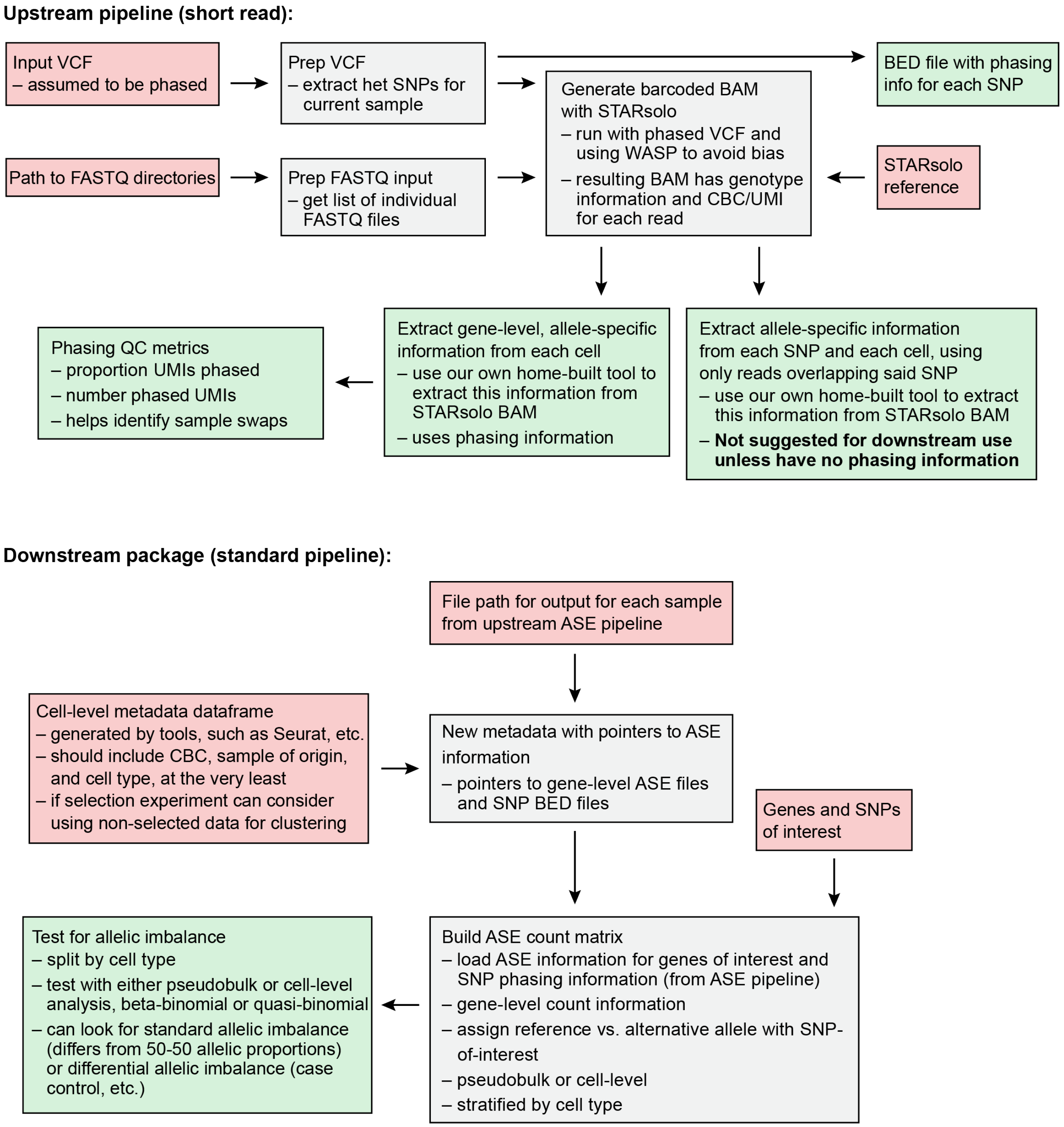

Fig. S11 Detailed computational pipeline.

More detailed outline of the computational processing pipeline starting from short-read data to generate ASE information and for downstream analysis (implemented in Nextflow).
